# Semiautomated segmentation of head and neck cancers in 18F-FDG PET scans: A just-enough-interaction approach

**DOI:** 10.1118/1.4948679

**Published:** 2016-05-18

**Authors:** Reinhard R. Beichel, Markus Van Tol, Ethan J. Ulrich, Christian Bauer, Tangel Chang, Kristin A. Plichta, Brian J. Smith, John J. Sunderland, Michael M. Graham, Milan Sonka, John M. Buatti

**Affiliations:** Department of Electrical and Computer Engineering, The University of Iowa, Iowa City, Iowa 52242; The Iowa Institute for Biomedical Imaging, The University of Iowa, Iowa City, Iowa 52242; and Department of Internal Medicine, The University of Iowa, Iowa City, Iowa 52242; Department of Electrical and Computer Engineering, The University of Iowa, Iowa City, Iowa 52242 and The Iowa Institute for Biomedical Imaging, The University of Iowa, Iowa City, Iowa 52242; Department of Radiation Oncology, The University of Iowa, Iowa City, Iowa 52242; Department of Biostatistics, The University of Iowa, Iowa City, Iowa 52242; Department of Radiology, The University of Iowa, Iowa City, Iowa 52242; Department of Electrical and Computer Engineering, The University of Iowa, Iowa City, Iowa 52242; Department of Radiation Oncology, The University of Iowa, Iowa City, Iowa 52242; and The Iowa Institute for Biomedical Imaging, The University of Iowa, Iowa City, Iowa 52242; Department of Radiation Oncology, The University of Iowa, Iowa City, Iowa 52242 and The Iowa Institute for Biomedical Imaging, The University of Iowa, Iowa City, Iowa 52242

**Keywords:** cancer segmentation, FDG PET imaging, graph-based segmentation, optimal surface finding, just-enough-interaction principle

## Abstract

**Purpose:**

The purpose of this work was to develop, validate, and compare a highly computer-aided method for the segmentation of hot lesions in head and neck 18F-FDG PET scans.

**Methods:**

A semiautomated segmentation method was developed, which transforms the segmentation problem into a graph-based optimization problem. For this purpose, a graph structure around a user-provided approximate lesion centerpoint is constructed and a suitable cost function is derived based on local image statistics. To handle frequently occurring situations that are ambiguous (e.g., lesions adjacent to each other versus lesion with inhomogeneous uptake), several segmentation modes are introduced that adapt the behavior of the base algorithm accordingly. In addition, the authors present approaches for the efficient interactive local and global refinement of initial segmentations that are based on the “just-enough-interaction” principle. For method validation, 60 PET/CT scans from 59 different subjects with 230 head and neck lesions were utilized. All patients had squamous cell carcinoma of the head and neck. A detailed comparison with the current clinically relevant standard manual segmentation approach was performed based on 2760 segmentations produced by three experts.

**Results:**

Segmentation accuracy measured by the Dice coefficient of the proposed semiautomated and standard manual segmentation approach was 0.766 and 0.764, respectively. This difference was not statistically significant (*p* = 0.2145). However, the intra- and interoperator standard deviations were significantly lower for the semiautomated method. In addition, the proposed method was found to be significantly faster and resulted in significantly higher intra- and interoperator segmentation agreement when compared to the manual segmentation approach.

**Conclusions:**

Lack of consistency in tumor definition is a critical barrier for radiation treatment targeting as well as for response assessment in clinical trials and in clinical oncology decision-making. The properties of the authors approach make it well suited for applications in image-guided radiation oncology, response assessment, or treatment outcome prediction.

## 1. INTRODUCTION

### 1.A. Background

FDG PET/CT has become an essential tool for clinical management of head and neck (H&N) squamous cell carcinoma (SCC).^1^ This disease typically originates from the normal squamous mucosa that lines the open air spaces in the H&N region. Cancers are most often caused by irritants and carcinogens from cigarette smoke, alcohol, and chewing tobacco although more recent studies also show a role for human papilloma virus (HPV).^2,3^ Once cancerous, these mucosal neoplasms have access to associated lymphatic drainage, leading to local/regional spread of the disease to neck lymph nodes. Staging for these cancers is defined by the size and invasion pattern of the primary site cancer (*T* stage) as well as the presence, size, and location of regional nodes (*N* stage).^4^ More rarely these cancers spread to regions beyond the H&N region, which can be defined as metastases (*M* stage).

TNM staging is an important determinant of both prognosis and oncologic treatment decision-making for every patient.^4^ Because of limitations of physical exam, imaging has rapidly become a critical part of staging. CT was the initial imaging modality of choice^5–8^ and provided volumetric anatomic assessment based on contrast uptake into tumor versus normal mucosa. The size of local-regional lymph nodes could readily be determined and lymph nodes larger than 1.5 cm were more likely involved in cancer. Unfortunately, CT imaging was not very sensitive with limited involvement of the cancer. The advent of PET/CT provided a more sensitive imaging tool that detected cancer based on the metabolic differences in glucose metabolism with tumors showing greater FDG uptake than normal tissues. FDG PET/CT is an effective tool for diagnosis and is effective in accurately detecting sites of cancer involvement and thereby improves the accuracy of staging^1,9^ and helps define areas for surgical excision and radiation therapy. FDG PET/CT has thus become the most important staging and therapy-planning tool for H&N cancers. Finally, the ultimate success of the treatment can be assessed with this more sensitive tool, although it requires a sufficiently long interval (generally 2–3 months) after radiation and/or surgery to allow the inflammation of treatment to abate.^10^ FDG PET/CT is now a standard in many centers for staging, clinical decision-making, and response assessment.^11^

Despite the advances associated with FDG PET/CT use, the previously described applications are mostly based on standard visual inspection of images with only limited use of quantitative indices. If quantification is employed, the most commonly used metrics to characterize lesions are SUVmax, SUVmean, metabolic tumor volume (MTV), and total lesion glycolysis (TLG).^12,13^ PERCIST is an approach that has proposed another index, SUVpeak (i.e., the maximum average activity in a 1 cm sphere inside the tumor of interest) to assess response to therapy.^14^

The work presented here focuses on improvements that can be achieved by employing more automated and therefore more consistent approaches to quantitative analysis of tumors and associated lymph nodes. We argue that highly automated segmentation algorithms can provide greater accuracy and consistency in defining radiotherapy treatment volumes and are important for quantifying responses to therapy.

### 1.B. Problem statement and requirements

**FIG. 1.**
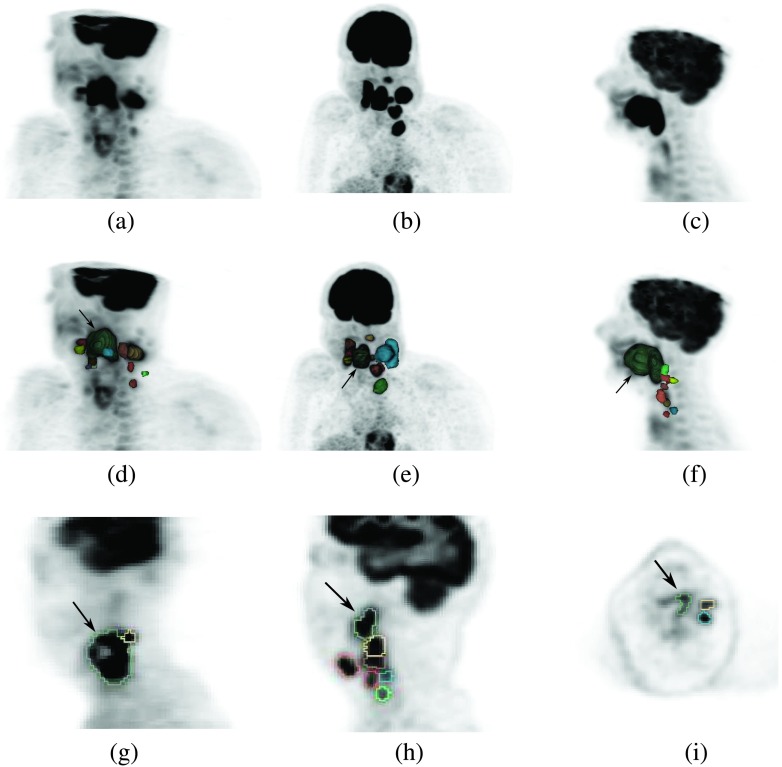
Examples depicting the complexity of HNC segmentation in FDG PET scans. [(a)–(c)] Volume rendering of PET scans, showing the primary cancer [arrow in (d)–(i)] and lymph nodes with uptake. [(d)–(f)] Rendering of segmentations of individual lesions shown in (a)–(c) combined with a volume rendering of the corresponding PET volume. [(g)–(i)] Examples of PET cross sections with outlined tumor segmentations. (g) Necrotic primary tumor with metabolically active adjacent lymph node. [(h) and (i)] Cases with multiple lesions and varying degrees of tracer uptake.

Segmenting H&N lesions in PET images is demanding, as demonstrated by the examples shown in Fig. 1, which are part of our evaluation set (Sec. 3.A). Also, for applications like outcome prediction, it is not *a priori* known if segmenting and quantitatively describing the primary tumor only is sufficient or if all lesions need to be quantified individually. Thus, to answer this question, all lesions need to be segmented individually and can be later combined for comparison. Specifically, our requirements for a head and neck cancer (HNC) segmentation approach are as follows.

• The segmentation method must offer the ability to individually segment lesions with varying contrast that are in close proximity to each other or to normal structures with FDG uptake, which is especially important for HNC FDG PET imaging (Fig. 1).
• The segmentation approach must allow physicians to segment lesions in a time-efficient manner, as required for clinical use. Note that due to the complexity of HNC FDG PET images, the (correct) interpretation of scans (i.e., locating lesions, differentiating between normal and abnormal structures, etc.) is already time intensive, and therefore, this point becomes even more important for this specific application.
• The method must be intuitive to use. Also, in the case of semiautomated segmentation methods, it must allow physicians to make corrections to semiautomated segmentation results as deemed appropriate.
• The approach must offer good agreement with manual segmentation, which is the de facto standard.
• The method must offer better consistency to reduce intra- and interoperator variability. This is especially important for applications like radiation treatment planning, where operator induced variability can compromise outcome.

### 1.C. Related work

The definition of the gross tumor volume (GTV) or calculation of FDG uptake metrics like average SUV, MTV, or total glycolytic volume (TGV) requires the segmentation of target structures like primary tumors and metabolically active lymph nodes in volumetric FDG PET images. For PET image segmentation, a number of approaches have been proposed, including adaptive thresholding,^15^ “standard” region growing,^16^ a combination of adaptive region-growing and dual-front active contours,^17^ *k*-means-based partitioning into tumor and background regions,^18^ a gradient-based method,^19^ a combination of two segmentation methods to reduce inconsistencies,^18^ and fuzzy locally adaptive Bayesian (FLAB) method.^20^ For an overview of published methods, the reader is referred to the review by Foster *et al.*^21^

Recently, a number of graph-based segmentation methods have been proposed for PET image segmentation, and a summary is given in Table I. Similarly as in other medical image analysis applications,^22^ these methods show great promise and represent a quite powerful segmentation framework. However, formulating a suitable graph-based cost function for HNC segmentation (Fig. 1) is not straightforward due to potentially contradicting/conflicting demands/requirements (e.g., lesion with inhomogeneous uptake vs. lesions in close proximity). Such an issue can be avoided by utilizing graph-segmentation algorithms in an interactive fashion, allowing the user to contribute expert knowledge, if needed. For example, in case of random walks and graph cuts, the user can add additional object and background seeds to alter the segmentation result. However, these changes can affect the segmentation globally, which can lead to unexpected effects, making segmentation refinement unintuitive. In contrast, we have developed approaches that follow the just-enough-interaction (JEI) principle for segmentation of livers,^23^ lymph nodes,^24^ and lungs in 3D (Ref. 25) and 4D (3D inspiration and expiration scan)^26^ MDCT scans as well as for segmentation of IVUS data sets.^27^ The idea behind this principle is that the user can provide simple cues to guide the segmentation algorithm in an efficient and intuitive manner. Besides the belief that the general JEI principle can offer improved performance in PET image analysis, no such approach for PET image segmentation has been available so far. Specifically, this paper represents the first demonstration that JEI strategy is suitable for this application domain.

### 1.D. Contribution of this work

In this paper, we present a clinically relevant JEI-based approach for FDG PET image segmentation, which utilizes a graph-based segmentation method. For this purpose, we transform the segmentation task into a suitable graph-based optimization problem. In addition, we introduce several efficient interaction approaches to enable the user to modify the initial segmentation result. To demonstrate the applicability and performance of our approach for the task of HNC segmentation, we evaluate our approach on a large cohort of FDG PET scans and compare its results to manual segmentation, the current clinical de facto standard for HNC segmentation in FDG PET volumes.

## 2. METHODS

We utilize a highly automated optimal surface segmentation (OSS) approach, which is a variant of the LOGISMOS (layered optimal graph image segmentation of multiple objects and surfaces) segmentation framework,^33^ for the segmentation of uptake in FDG PET volumes. OSS was introduced by Li *et al.*^34^ The basic idea behind this approach is to formulate a segmentation problem as a graph-based optimization problem, which enables finding an optimal solution (i.e., segmentation surface) according to the utilized cost function. For more information on OSS, the reader is referred to Li *et al.*^34^

In Subsections 2.A–2.E, the approach for lesion segmentation in FDG PET volumes is described in detail. First, we introduce the main segmentation approach (Secs. 2.A–2.C). Second, we outline several additional segmentation modes, which allow effective handling of frequently occurring situations like lesions in close proximity (Sec. 2.D). Third, we describe an approach that enables the user to efficiently refine segmentations (Sec. 2.E), if needed.

### 2.A. Graph construction

To construct a graph that represents the segmentation problem, the user first needs to indicate a lesion to be segmented. For this purpose, the user specifies a rough center point *ce_k_* inside of lesion *k*. This can be done in two ways: (a) the user-selected point *ce*_*k*_user__ is directly utilized as *ce_k_* or (b) *ce*_*k*_user__ can be automatically recentered to the highest uptake voxel within a search radius of 7 mm from *ce*_*k*_user__. Option (b) typically leads to more consistent results because a property of the PET image *I* is utilized to adjust the location of the center point. Therefore, it is used as the default setting, which can be changed by the user. However, recentering may cause problems, for example, when segmenting lesions with necrotic centers.

**FIG. 2.**
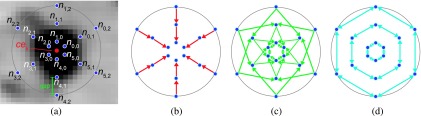
Graph construction for OSS. (a) Centerpoint *ce_k_* and utilized node structure; the spherical mesh is symbolized by a circle. For each node, a cost is assigned. (b) Intracolumn edge structure (red) with infinite capacity, pointing toward the center. [(c) and (d)] Intercolumn edge structure. (c) The hard smoothness constraint is implemented by introducing edges with infinite capacity shown in green. (d) The soft smoothness constraint is implemented by introducing bidirectional edges with low finite capacity shown in cyan. (See color online version.)

Based on *ce_k_*, a graph structure is generated (Fig. 2). For this purpose we assume that the lesion to be segmented is roughly spherical in shape, which is frequently the case for lesions in the H&N area. For more complex shaped lesions, additional center points can be used, and individual segmentation results will be automatically combined with a logical OR operation. A graph *G_k_* = (*V*, *E*), consisting of nodes *V* and edges *E*, is constructed by placing a spherical mesh with radius *r* and *n*_column_ evenly spaced mesh vertices *p_i_* with *i* ∈ {0, 1, …, *n*_column_ − 1} centered around *ce_k_*. The volume (voxels) inside the spherical mesh will be denoted as *M*_region_. Then, columns with sample points (nodes) are introduced between *ce_k_* and mesh vertices *p_i_*. The spacing between nodes is *gap*, resulting in *n*_node_ nodes that represent sample points of the physical volume [Fig. 2(a)]. The node *n*_*i*,0_ is closest to the center, while node *n*_*i*,*n*_node_−1_ is furthest away from the center. All remaining nodes are placed in order between them. In addition to nodes, several edges are added to represent the segmentation problem. First, for all nodes on a column, the edges {*n*_*i*,*j*_, *n*_*i*′,*j*−1_} with infinite capacity are added to *E* if *j* ≠ 0 to ensure that only one node can be selected on a column^34^ [Fig. 2(b)]. Second, for all nodes *j* and *j*′, where *j*′ = max(*j* − *sc*, 0) on every pair of adjacent columns *i* and *i*′, edges {*n*_*i*,*j*_, *n*_*i*′,*j*′_} and {*n*_*i*′,*j*_, *n*_*i*,*j*′_} with infinite capacity are added to *E* to implement a hard smoothness constraint^34^ [Fig. 2(c)]. Thus, when solved, the nodes of two adjacent surface points on columns cannot be more than *sc* nodes apart. Third, a soft smoothness constraint is included by adding edges {*n*_*i*,*j*_, *n*_*i*′,*j*_} and {*n*_*i*′,*j*_, *n*_*i*,*j*_} with capacity *sp* to *E*^35^ [Fig. 2(d)]. Consequently, for a possible solution, the cost is increased by *sp* times the difference in *j* between surface nodes on a neighboring column.

Based on experiments performed on a small set of PET volumes dedicated for algorithm development, we found that *r* = 60.0 mm, *n*_column_ = 1026, gap = 1.0 mm (resulting in *n*_node_ = 60), *sc* = 5, and *sp* = 0.005 work well for our application.

### 2.B. Cost function design

**FIG. 3.**
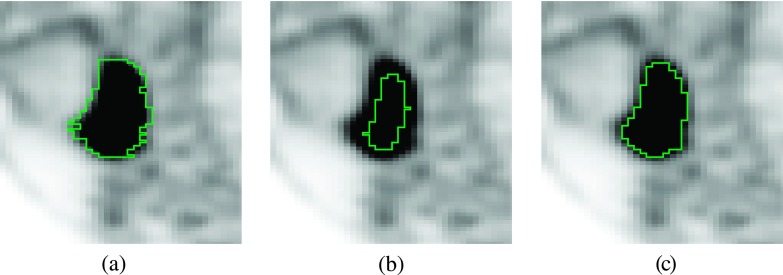
Comparison of PET segmentation approaches. The same sagittal cross section is shown for all segmentations. (a) Manual slice-by-slice segmentation result. (b) Result of a 50% isocontour segmentation approach. (c) Result of proposed graph-based segmentation method.

For graph-based segmentation, nodes *n*_*i*,*j*_ that represent the object boundary should incur low costs, while nodes that represent the selected object (uptake region), adjacent objects, or background should incur high costs to make it less likely that the object surface (mesh) passes through these nodes. To generate node costs, uptake values *up*(*n*_*i*,*j*_) at corresponding spatial node locations are sampled from the PET volume by means of linear interpolation and subsequently converted into node costs *c*_base_(*n*_*i*,*j*_). This conversion is based on the analysis of local image properties around the center point *ce_k_*, which are robustly estimated. Our cost function design is based on an adaptive strategy to mimic the typical tracing preference of radiation oncologists [Fig. 3(a)]. The boundary drawn by the radiation oncologist [Fig. 3(a)] almost reaches voxels with background uptake, whereas a typical segmentation approach based on the isocontour corresponding to 50% of the maximum lesion uptake-to-background uptake ratio has boundary voxels with much higher uptake values [Fig. 3(b)]. In this context, we observed that there is no linear relation between the average uptake value at the boundary of the manual segmentation and the maximum uptake to background uptake ratio. Also, note that the segmentation boundary appears “noisy” in the sagittal cross section depicted in Fig. 3(a), because the manual segmentation was performed in an axial slice-by-slice fashion. The proposed approach to cost function calculation addresses all these issues [Fig. 3(c)]. All specific design decisions and parameter selection are based on experiments performed on the same set of PET volumes that have been used for optimizing graph construction parameters (Sec. 2.A).

#### 2.B.1. Local image properties

**FIG. 4.**
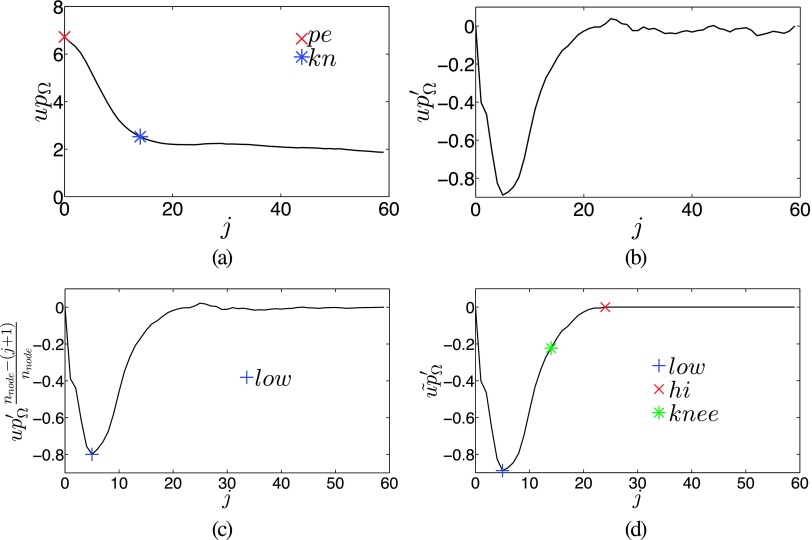
Calculation of local image statistics. (a) Plot of *up*_Ω_ with points of interest *pe* and *kn*. (b) The gradient of *up*_Ω_. (c) The gradient with added center bias to detect *j*_low_. (d) Deriving *j*_hi_ from ${\overset{˜}{up_{\Omega }}}^{\prime }$. Points on the curve that are labeled with low and hi mark the location of indices *j*_low_ and *j*_hi_, respectively.

Based on our assumption of roughly spherical object shape, we introduce the notion of a shell to robustly measure local uptake parameters. A shell Ω_*j*_ is the set of all nodes with the same node level j: Ω_*j*_ = {*n*_*i*,*j*_ ∣ *i* = 0, …, *n*_column_ − 1}. The average uptake for each shell Ω_*j*_ is robustly estimated by *up*_Ω_(*j*) = median{*up*(*n*_*i*,*j*_) ∣ *n*_*i*,*j*_ ∈ Ω_*j*_}. A typical plot of *up*_Ω_(*j*) as a function of *j* is given in Fig. 4(a). Specifically, we are interested in finding the “peak” *pe* and the “knee” *kn* uptake values [Fig. 4(a)], which will be utilized for determining a threshold *Th* that is used for calculating *c*_base_(*n*_*i*,*j*_). The peak *pe* is given by $pe=\underset{j=0,\ldots ,\underset{\mathrm{node}}{n}-1}{\max}\left(up_{\Omega }\left(j\right)\right)$ and represents the maximum uptake in a shell. The knee *kn* is the approximate uptake value at which the object fades into the background, but is not intended to be the background itself. To find *kn*, the gradient $up_{\Omega }^{\prime }\left(j\right)=up_{\Omega }\left(j+1\right)-up_{\Omega }\left(j-1\right)$ is calculated for *j* = 1, …, *n*_node_ − 2 [Fig. 4(b)]. Then the index of the node representing the steepest descent in *up*_Ω_ is found with $j_{\mathrm{low}}=\arg\;\min_{j=1,\ldots ,n_{\mathrm{node}}-2}\left\{\left(n_{\mathrm{node}}-\left(j+1\right)\right)/n_{\mathrm{node}}up_{\Omega }^{\prime }\left(j\right)\right\}$ [Fig. 4(c)]. The linear center bias term (*n*_node_ − (*j* + 1))/*n*_node_ helps dealing with some rare, occasional cases of one large or several small outside objects with uptake appearing in the shell profile. To find the point where the shell uptake transitions to background values, we utilize a modified version of $up_{\Omega }^{\prime }$, such that it never decreases if *j* > *j*_low_

$${\overset{˜}{up_{\Omega }}}^{\prime }\left(j\right)=\{\begin{array}{ll} up_{\Omega }^{\prime }\left(j\right), & \text{if}\;j\leq j_{\mathrm{low}}\\ \max\left(up_{\Omega }^{\prime }\left(j\right),\;{\overset{˜}{up_{\Omega }}}^{\prime }\left(j-1\right)\right), & \text{otherwise} \end{array}.$$ (1)

The node index where *up*_Ω_ starts leveling off is *j*_hi_ [Fig. 4(d)] with ${\overset{˜}{up_{\Omega }}}^{\prime }\left(j_{\mathrm{hi}}\right)=\min\left(0,\underset{j=\underset{\mathrm{low}}{j},j_{\mathrm{low}}+1,\ldots ,n_{\mathrm{node}}-1}{\max}\left({\overset{˜}{up_{\Omega }}}^{\prime }\left(j\right)\right)\right)$. The knee node index is found by evaluating $j_{\mathrm{knee}}=\arg\;\min_{j=j_{\mathrm{low}},j_{\mathrm{low}}+1,\ldots ,j_{\mathrm{hi}}}|{\overset{˜}{up_{\Omega }}}^{\prime }\left(j\right)-\left(0.75\;{\overset{˜}{up_{\Omega }}}^{\prime }\left(j_{\mathrm{hi}}\right)+0.25\;{\overset{˜}{up_{\Omega }}}^{\prime }\left(j_{\mathrm{low}}\right)\right)|$ and *kn* = *up*_Ω_(*j*_knee_).

#### 2.B.2. Threshold calculation

Once the peak and knee are determined, the threshold *Th* can be calculated evaluating *Th* = *kn* + *Th*_%_(*pe* − *kn*) with

$$Th_{\%}=0.8e^{-0.15\;\gamma^{1.5}}.$$ (2)

**FIG. 5.**
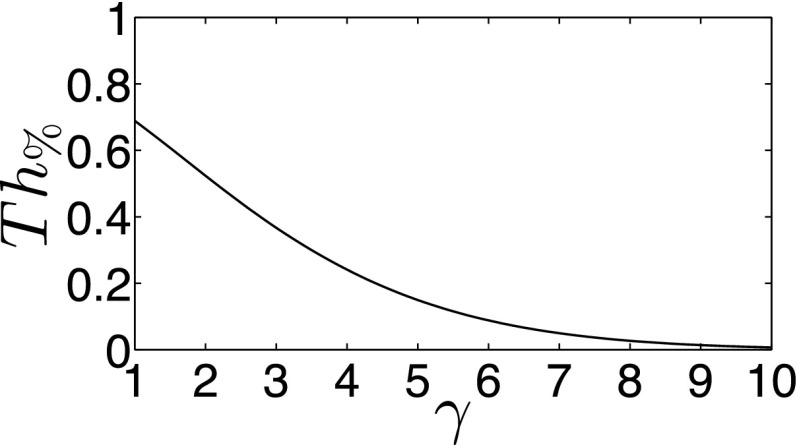
Plot of *Th*_%_ as a function of *γ*.

A plot of Eq. (2) as a function of the ratio *γ* = *pe*/*kn* is provided in Fig. 5, and the rationale behind this design is as follows. If *γ* = 2, roughly a 50% threshold is used. For larger *γ*-values (e.g., *pe* ≫ *kn*), the *Th*_%_ will be lower than 50%, and for smaller *γ*-values, *Th*_%_ will be increased moderately. With this empirical design, the typical tracing behavior of radiation oncologists is mimicked; higher uptake values above background are included to form a perceived safe tumor margin definition (Fig. 3). High acceptance (i.e., avoiding the urge of users to manually edit/postprocess segmentations that were generated by an algorithm) of computer-generated thresholds/segmentations is important to achieve lower inter- and intraobserver variability. Furthermore, Eq. (2) can be tailored to specific needs or applications. For example, variants based on some standard threshold methods can be used as an alternative to focus more on volume estimation than treatment. Some examples are *Th*_%_ = 0.4 and *Th*_%_ = 0.5, which are based on typical 40% and 50% values of maximum thresholds that are fairly common. Also, note that during calculation of *Th*, the most inner and outer shells are not considered because they represent extreme solutions that are not of interest.

#### 2.B.3. Cost function

**FIG. 6.**
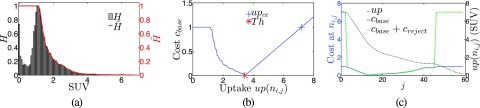
Cost function design. (a) Histogram *H* of a region around a typical lesion with corresponding envelope function $\overset{˜}{H}$. (b) Example of a cost function; the individual components are clearly visible. The left part follows $\overset{˜}{H}$, while the right part is linear. The lowest point where both parts meet is at *Th*. (c) Example of a typical cost profile with and without *c*_reject_ on a single column *i*, as a function of *j*, paired with the uptake along the column.

Once the threshold *Th* is found, the cost function *c*_base_(*n*_*i*,*j*_) is constructed as follows (Fig. 6). Essentially, *c*_base_ consists of the following three components:

**If***up*(*n*_*i*,*j*_) < *Th*: The cost $c_{\mathrm{base}}\left(n_{i,j}\right)=\overset{˜}{H}\left(up\left(n_{i,j}\right)\right)$ reflects the likeliness of the uptake to be part of the background. For this purpose, the image volume inside *M*_region_ is isotropically resampled, and a normalized image histogram *H* with max(*H*) = 1 is generated. $\overset{˜}{H}$ is then generated by taking the right-to-left monotonic increasing envelope function of *H* [Fig. 6(a)].
**If***up*(*n*_*i*,*j*_) = *Th*: The cost is defined as *c*_base_(*n*_*i*,*j*_) = 0.
**If***up*(*n*_*i*,*j*_) > *Th*: The cost is a linear function given by *c*_base_(*n*_*i*,*j*_) = (*up*(*n*_*i*,*j*_) − *Th*)/(*up*(*ce_k_*) − *Th*), which has the value of 0 at the threshold uptake and 1 at the center uptake.

The final cost function is given by *c*(*n*_*i*,*j*_) = *c*_base_(*n*_*i*,*j*_) + *c*_reject_(*n*_*i*,*j*_). The term *c*_reject_(*n*_*i*,*j*_) is added to reject trivial solutions or parts of adjacent, unrelated objects. It is defined as

$$c_{\mathrm{reject}}\left(n_{i,j}\right)=\{\begin{array}{ll} \mathrm{rej}, & \text{if}\;r_{1}\left(n_{i,j}\right)\;\text{or}\;r_{2}\left(n_{i,j}\right)\\ 0, & \text{else} \end{array},$$ (3)

with *r*_1_(*n*_*i*,*j*_) = *j* < *j*_min_, $r_{2}\left(n_{i,j}\right)=\left(j>j_{\min}\;\text{and}\;\underset{\overset{\prime }{j}=0,1,\ldots ,j}{\min}\left(up\left(i,j^{\prime }\right)\right)<\mathrm{median}\left(M_{\mathrm{region}}\right)\right)$, and *j*_min_ = 3. Therefore, the costs of nodes in close proximity to the center (*j* < *j*_min_) as well as nodes further away where the uptake has fallen below the median of spherical region *M*_region_ are increased by *rej* = 6, far above the typical cost range between 0 and 1. An example of a complete cost function profile is given in Fig. 6(c).

### 2.C. Segmentation

**FIG. 7.**
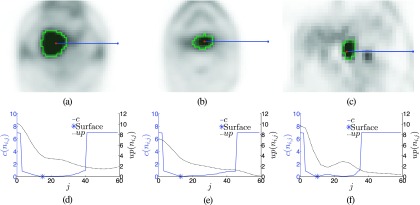
Examples of lesion segmentation results. [(a)–(c)] Segmentation results with a marked column along the *x*-axis. [(d)–(f)] Plots of the uptake and cost function corresponding to (a)–(c).

Once the graph is constructed and all costs have been determined, a globally optimal solution can be found in low degree polynomial time, as described by Li *et al.*^34^ The result is a set of nodes with exactly one node per column selected, which represents the segmentation boundary. To visually represent the segmentation result, the initial spherical triangle mesh can be utilized by moving mesh vertices to selected boundary nodes on the same column. Several examples of segmentations and their cost functions are shown in Fig. 7.

Once the user has produced a valid segmentation of lesions found in a PET volume, the meshes are converted to labeled volumes by means of voxelization, resulting in segmentations *S_k_*. Voxelization can lead to a result where some voxels are not within a 6-neighborhood of the main part of the lesion that includes *ce_k_*. Such voxels are removed. Also, the user has the option to close one-voxel-wide gaps between adjacent lesions (e.g., hot lymph nodes within a chain of nodes).

### 2.D. Segmentation options

Beyond the base algorithm (Secs. 2.A–2.C), there are alternative modes that can adapt the behavior of the algorithm to better handle frequently occurring situations that are ambiguous. Thus, the options enable the user to provide additional expert knowledge, which is utilized to modify the behavior of the algorithm accordingly. For our method, the following segmentation options were implemented, which are described in appendices. *Label Avoidance* prevents new lesion segmentation from overwriting existing segmentations (Appendix A). *Splitting* simplifies individually segmenting lesion in close proximity with similar uptake (Appendix B). *Necrotic Mode* simplifies the segmentation of necrotic lesions (Appendix C).

### 2.E. JEI-Based refinement approach

**FIG. 8.**
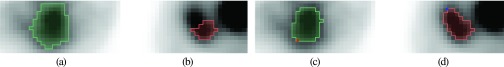
Examples of JEI-based segmentation refinement. (a) The segmentation surface expanded outward too much. (b) A part of the lesion was excluded. (c) The segmentation shown in (a) is corrected with one mouse click by using the global refinement option, affecting the whole boundary. (d) The segmentation shown in (b) is corrected with one mouse click by using local refinement, affecting only a local portion of the boundary. (See color online version.)

Designing a cost function that is appropriate for all possible situations is difficult. Consequently, while the above-described base algorithm will perform flawlessly for the majority of lesions found in H&N FDG PET scans, suboptimal results can occur in some cases. For practical applicability in clinical trials and routine care, it is important that even difficult cases can be processed with the same segmentation tool without major additional effort. Thus, we utilize the JEI principle to effectively deal with segmentation errors of the base algorithm. The basic idea behind JEI is that user input is kept at a minimum during refinement of a segmentation. Thus, instead of manually correcting (local) errors (i.e., user driven boundary delineation), the user provides only high-level input to the algorithm to correct the segmentation boundary by utilizing refinement modes of the approach. Also, an undo function is provided that allows the user to reverse a refinement step. Two examples of JEI-based refinement are given in Fig. 8, and a detailed description of refinement modes is given in the sections below.

#### 2.E.1. Global refinement

Global refinement changes the value of *Th* in order to modify the boundary of the segmentation. The user can place a point *R_Th_* at an image location where the lesion boundary should go through, and the corresponding uptake value is utilized as a new value for *Th*, which will change the cost function and thus the entire segmentation surface. In addition, the closest column to *R_Th_* is modified to force the result (surface) through the closest node to *R_Th_*, which will be called *n*_*i_Th_*,*j_Th_*_. This is accomplished by adding another cost change function

$$c_{Th}\left(n_{i,j}\right)=\{\begin{array}{ll} 1000, & \text{if}\;i=i_{Th}\;\text{and}\;j\neq j_{Th}\\ 0, & \text{else} \end{array}$$ (4)

to *c*_base_ during the calculation of the final cost *c*. As a result of this cost change, the entire graph-based optimization needs to be rerun. If the user is not satisfied with the result, he/she can repeat the process, which will override the previous settings. An example for a “single click” global refinement action is provided in Figs. 8(a) and 8(c). As can be seen, a single user provided point [red dot in Fig. 8(c)] is sufficient to update the whole object surface.

#### 2.E.2. Local refinement

Local refinement allows the user to correct cases where only a portion of the segmentation boundary needs to be corrected. This is accomplished as described below.

**FIG. 9.**
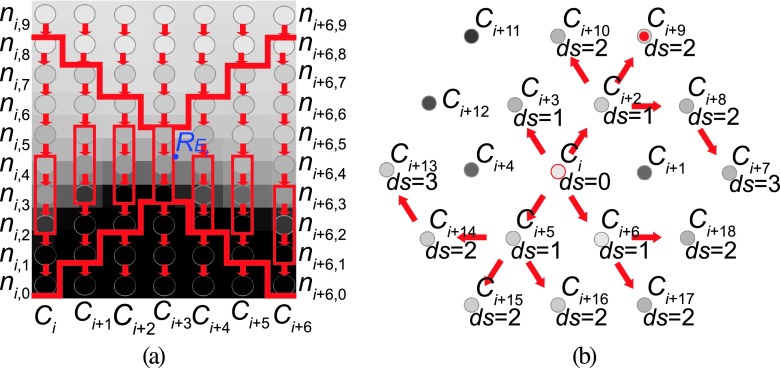
Examples illustrating local refinement. (a) In this example, the vector around *R*_*E_l_*_ on the column marked *C*_*i*+3_ is compared to vectors on adjacent columns within range to find the best match for the refinement. Note that the vector is smaller than in practice and for simplicity, only columns in a plane are shown. (b) Illustration of BFS to find neighboring columns whose costs need to be adapted. Note that in this case, comparisons are based on column *C_i_*.

(a) *User-interaction*. To start the local refinement process, the user needs to inspect the segmentation and, if needed, specify a surface point *R_L_*_*p*_ through which the correct surface should go [blue point in Fig. 8(d)]. Because the user can perform multiple local refinement interactions, the index *p* is used to keep track of them. The node closest to *R_L_*_*p*_ will be denoted as *n*(*i_L_*_*p*_, *j_L_*_*p*_).
(b) *Local search for similar columns*. Subsequent to finding *n*(*i_L_*_*p*_, *j_L_*_*p*_), neighboring columns of the surface segment to be altered are determined based on the similarity of the uptake pattern around *n*(*i_L_*_*p*_, *j_L_*_*p*_) (Fig. 9). For this purpose, a breadth-first search (BFS) on columns with a hard constraint of *ds*(*i*, *i_L_*_*p*_) ≤ 5 is performed. The function *ds*(*i*, *i_L_*_*p*_) represents the number of edges on the shortest path on selected columns (mesh vertices) between the surface mesh vertices *a* and *b* (graph geodesic) that are associated with columns *i* and *i_L_*_*p*_, respectively. An uptake pattern on a column is considered similar if $smc\left(n_{i,j},n_{{i_{L}}_{p},{j_{L}}_{p}}\right)=\overset{3}{\underset{k=-3}{\sum }}|up\left(n_{i,j+k}\right)-up\left(n_{{i_{L}}_{p},{j_{L}}_{p}+k}\right)|<th_{sim}$ and |*j* − *j_L_*_*p*_| ≤ *ds*(*i*, *i_L_*_*p*_) is fulfilled with $th_{sim}=0.05\overset{3}{\underset{k=-3}{\sum }}|up\left(n_{i,j+k}\right)|$. Because the BFS can leave “holes” in the set of selected columns, left out columns are added in a “closing” step; columns that were skipped during BFS due to dissimilarity are included, if two thirds of its immediate neighbors were selected by *smc*. Finally, for all selected columns with *i*≠*i_L_*_*p*_, the node with the highest profile similarity is stored in the set Ψ_*p*_.
(c) *Modification of costs*. To locally refine the segmentation result, the costs on column *i_L_*_*p*_ itself and selected neighboring columns in set Ψ_*p*_ are modified. On column *i_L_*_*p*_, the cost update function is given by
  $${c_{L}}_{p}\left(n_{{i_{L}}_{p},j}\right)=\{\begin{array}{ll} 1000, & \text{if}\;j\neq {j_{L}}_{p}\\ 0, & \text{else} \end{array}.$$ (5)
  For all nodes of columns that include a node $n_{{\overset{˜}{i}}_{p},{\overset{˜}{j}}_{p}}\in \Psi_{p}$, the function ${c_{L}}_{p}\left(n_{{\overset{˜}{i}}_{p},j}\right)=\mathrm{notch}\left(j,{\overset{˜}{j}}_{p},3,ds\left({\overset{˜}{i}}_{p},{i_{L}}_{p}\right)\right)$ is utilized; for the definition of function notch see Eq. (A2) in Appendix A. Thus, the cost is decreased around the target. The decrease is narrow in close proximity to column *i_L_*_*p*_, but becomes much wider further away. For all other nodes, *c_L_*_*p*_ is equal to zero. The motivation behind this design is as follows. First, it forces the boundary to pass trough *n*_*i_L_*_*p*_,*j*_. Second, it models uncertainty further away from the user specified refinement point. Third, it enables a smooth transition to the unchanged portions of the surface.
(d) *Calculating a new solution*. After adding *c_L_*_*p*_ to the original cost function *c*, a new solution is calculated and the segmentation result is displayed. Calculating a new solution can be done efficiently by building on the previously calculated one, as outlined by Boykov and Jolly.^36^

Multiple local refinement points can be utilized for a lesion, each resulting in a cost change, which are accumulated to update *c*. The only exceptions are columns directly associated with user-selected refinement points, which will not have their costs further changed.

## 3. METHOD VALIDATION

### 3.A. Image data

**FIG. 10.**
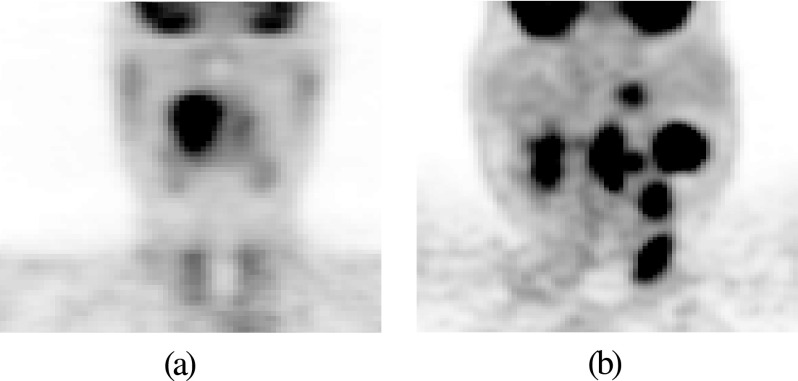
Range of complexity of utilized H&N PET image data. (a) Case with low complexity (single primary tumor) and (b) a case with high complexity with primary cancer and multiple hot lymph nodes in close proximity.

For method validation, 60 PET/CT scans from 59 different subjects with H&N cancer were utilized, which were acquired with different scanners and reconstruction parameters (Table II) between 2004 and 2008. Out of this set, 59 scans were performed pretreatment, and for one patient, an additional post-treatment scan with uptake was included. All subjects were injected with 370 MBq ± 10% of [F-18]FDG with an uptake time of 90 min ± 10%. In all cases subjects were fasted for >4 h and had blood glucose <200 mg/dl. Because of the interest in the H&N region, patients were imaged with arms down, and CT-based attenuation correction was performed. All reconstructions were performed with 2D OSEM iterative algorithms (Table II). Siemens Biograph 40 PET images were reconstructed onto a 168 × 168 pixel image matrix, while Siemens Biograph Duo PET and GE Medical Systems Discovery LS images were reconstructed onto a 128 × 128 pixel image matrix. The number of axial image slices ranged from 191 to 545. The complexity of cases to segment covered a wide spectrum, ranging from simple to complex (Figs. 10 and 1). Primary cancers included the following anatomical regions: base of tongue, oropharynx, pyriform sinus, tonsil, hypopharynx, and nasopharynx. The cases had varying TNM stage, but no metastasis. An experienced radiation oncologist (JMB) inspected all scans and identified primary tumors and all positive (hot) lymph nodes. Overall, the PET scans include a total of 230 different lesions with 59 primary tumors and 171 lymph nodes, and the average number of lesions was 3.83/scan. All scans were assessed in terms of complexity and classified into the following categories: low, med, and high.

### 3.B. Experimental setup

To assess the performance of our *semiautomated* algorithm, it was implemented as an extension for 3D Slicer,^37^ a multiplatform, free, and open source software package for visualization and medical-image computing. In addition, the *manual* segmentation (2D drawing) tools offered by 3D Slicer were used for comparison.

Three physicians (experts) with different levels of radiation oncology experience (one faculty professor—JMB and two physician residents with instructions—KAP and TC) participated in our validation experiment. Before the start of the experiment, all experts were trained on both segmentation methods and tools, using a separate set of ten H&N PET scans.

For validation, the 60 PET scans described in Sec. 3.A were randomly divided into three sets of 20 scans each. The partitioning was stratified such that each set contains approximately the same range of case complexities. These three sets were processed sequentially in four processing steps. In each step, the complete set of 20 PET scans was segmented in a random sequence by alternating the use of semiautomated and manual segmentation methods such that after two steps, all 20 cases in a set were processed with both methods by each physician. To assess intraoperator variability, two additional steps were performed by all three experts. Thus, all 230 lesions present in the 60 PET scans partitioned into three sets were segmented four-times by three experts, resulting overall in 2760 segmentations. Half were done by using the semiautomated (JEI) method and half with the standard manual segmentation method. For both methods, PET scans were loaded into the segmentation software using a default display setting, consisting of a window of 6 SUV and level of 3 SUV. The experts were allowed to change this setting if deemed necessary for the segmentation task at hand (e.g., chain of active lymph nodes with similar uptake).

**FIG. 11.**
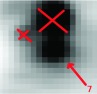
Example of an indicator image provided to experts to specify what lesions should be segmented and the label (seven in this case) that should be assigned.

For segmentation, a set of indicator images was provided to the experts that roughly identified the object to be segmented and the corresponding object label to be used (Fig. 11) for each lesion to avoid differences in the interpretation of the PET scans. The indicator images were generated by an experienced radiation oncologist (JMB) with access to medical patient records.

### 3.C. Independent reference standard and quantitative indices

(a) *Segmentation accuracy*. Due to the lack of a ground truth for lesions, the following approach was used to form an independent reference for comparison. For a given expert, the four manual segmentations performed by the two other experts were combined to produce a segmentation reference by utilizing the Simultaneous Truth and Performance Level Estimation (STAPLE) algorithm proposed by Warfield *et al.*^38^ To assess segmentation accuracy, the Dice coefficient was computed between each expert’s observed and reference segmentations. Given two segmentations, *A* and *B*, the Dice coefficient is given by *D* = (2|*A*∩*B*|)/(|*A*| + |*B*|). Coefficient values close to one indicate high agreement, and values close to zero indicate low agreement.
(b) *Segmentation agreement*. For the assessment of inter- and intraoperator segmentation agreement, the following approach was used. To assess intraoperator agreement, the Dice coefficient between trial one and two with a given method was calculated. To measure interoperator agreement, Dice coefficients for a given method were calculated for all user pairs within each trial.
(c) *Time and user effort*. For each user, the time required to segment all lesions in a given PET scan was recorded for both methods. Additionally, for the semiautomated approach, the number of actions (specifying a center point for segmentation, refinement operations, etc.) including and excluding discarded actions was recorded and analyzed.

### 3.D. Statistical analysis of performance indices

Statistical analysis was performed on results from the tumor segmentations. Reported Dice coefficient means, 95% confidence intervals, and *p*-values were obtained with linear mixed effects regression models. Random effects were included in the models for experts and patients in order to account for repeated segmentation within the two factors. Estimated intra- and interoperator variability in segmentation accuracy Dice coefficients are reported as standard deviations. Intraoperator variability measures the amount of variation in the trial one and two segmentations about their average. Interoperator variability measures the variation in trial one and two averages across experts.

## 4. RESULTS

### 4.A. Segmentation accuracy

For both methods, the average Dice coefficient is given in Table III. The Dice coefficients were not found to be statistically different (*p* = 0.2145). Also, Table III provides summaries of the intra- and interoperator standard deviation of segmentation accuracy assessed with the Dice coefficient. The provided 95% confidence intervals (CIs) show that intra- and interoperator standard deviations are significantly lower for the semiautomated method compared to the manual segmentation approach.

### 4.B. Segmentation agreement

**FIG. 12.**
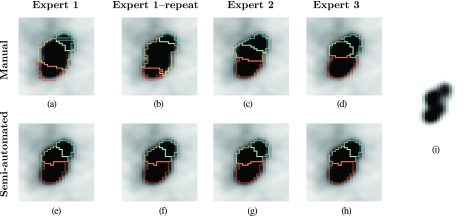
Example of intra- and interoperator segmentation agreement for manual and semiautomated segmentation methods. [(a)–(d)] Manual slice-by-slice segmentation results. [(e)–(h)] Semiautomated full 3D segmentation results. (i) Same PET image as in images (a)–(h), but with a different grey-value transfer function, showing uptake peaks corresponding to individual lymph nodes in close proximity.

Table IV summarizes results for intra- and interoperator segmentation agreement analysis. In both cases, the semiautomated method shows a significantly higher agreement compared to the manual method. Figure 12 depicts typical examples of intra- and interoperator variation in HNC segmentation.

### 4.C. Time and user effort

**FIG. 13.**
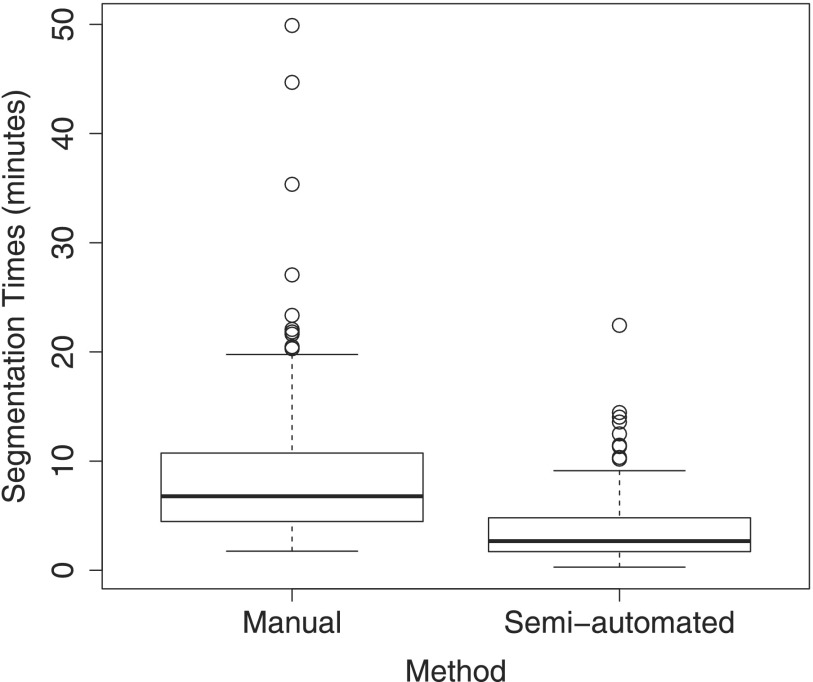
Boxplots of operator segmentation times for the manual and semiautomated segmentation methods per PET scan.

Table V and the box-plots provided in Fig. 13 summarize the time required for producing manual and semiautomated PET segmentations per HNC PET scan. The 95% confidence intervals in Table V show that the proposed semiautomated method is significantly faster than its manual counterpart. Note that while the average time for segmenting all lesions (average: 3.83 lesions) with our semiautomated method in a PET scan was 3.74 min, the segmentation algorithm itself runs at interactive speeds. Thus, almost all of the reported time is spent by the user on understanding the scene and inspecting produced segmentations.

**FIG. 14.**
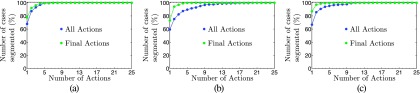
Plots showing the accumulative number of cases completely segmented in dependence of number of user actions required for the semiautomated method for experts one, two and three. “All actions” denotes the number of actions actually performed by the expert, and “final actions” denotes the user actions that were actually required to perform the segmentations (i.e., undone actions are not counted).

Table VI provides statistics about the number of actions required by experts to perform a semiautomated segmentation of all lesions in a PET scan. The plots in Fig. 14 show the accumulative percentage of cases that were completely segmented in dependence of the number of actions required. Differences between used (all) and actually necessary (final) actions in Table VI and Fig. 14 indicate that the user effectiveness can be further increased with additional training.

## 5. DISCUSSION

### 5.A. Performance

Our JEI graph-based segmentation approach offers a high degree of automation, requires little user interaction, and enables clinically practical and efficient computer-aided segmentation refinement. Compared to a manual segmentation approach, this combination results in a versatile segmentation tool that showed equivalent average segmentation error with significantly reduced standard deviation. The performed assessment of user agreement showed significantly higher intra- and interoperator consistency for the semiautomated approach compared to manual segmentation. This can be seen by the example provided in Fig. 12; the manual segmentation approach leads to considerable variation in segmentations produced by the same user as well as across users, whereas little variation is observed between the segmentations produced with the semiautomated method. Thus, the approach meets the requirements defined in Sec. 1.B.

Despite the relatively short training phase, all three experts were able to produce valid segmentations of target lesions in all utilized PET scans. However, as Table VI and Fig. 14 show, training of users is important to achieve good efficiency. Based on the plots shown in Fig. 14, we estimate that more than 90% of the 230 lesions can be segmented with one or two user input actions (mouse clicks), which will likely reduce the required user interaction time (Table V and Fig. 13) even further.

### 5.B. Current limitations

As can be seen from Fig. 14, in some rare cases, more than ten user actions were necessary to produce a segmentation of a lesion. This issue can be addressed by adding suitable segmentation modes and/or refinement tools that enables the user to more efficiently handle such cases.

While the results presented in Sec. 4 are very promising in terms of segmentation error, segmentation agreement, and interaction time, it does not offer insight into the impact of different PET image reconstruction methods on segmentation results. Thus, we plan to study this aspect by utilizing PET phantoms in the near future. Harmonization of image acquisition and quality assurance is another critical component of assuring optimal clinical trials, target definitions, and response assessment that is also being addressed nationally.^39^

In the current implementation, only the PET image information of PET-CT scans is utilized. However, the chosen graph-based LOGISMOS framework is well suited for expanding our approach to segmenting PET and CT volumes at the same time. In addition, the method can be adapted for segmenting dynamic PET (volume and time) scans. Also, by defining a suitable cost function, our segmentation approach can be adapted for segmenting PET scans with tracers other than FDG.

### 5.C. Impact

Target or lesion definition is a critical component for both radiation therapy treatment planning and delivery as well as for response assessment of tumors after treatment. Our current standard involves a process wherein the radiation oncologist or radiologist manually outlines a tumor or lesion. This is inherently both time consuming and relatively inconsistent because of the nature of both human perception and limits of human dexterity. Algorithmic tools have the capacity to speed the process and at the same time improve consistency.

While concerns exist regarding automation of critical treatment-determining steps and decision-making metrics, the ability to improve consistency is unquestionably paramount, particularly when considering clinical trials in which multiple institutions and investigators will determine targets and hence outcomes. The ability to substantially improve consistency enables better comparisons of tumor control probabilities. Although substantial quality assurance efforts within the radiation therapy community have been extremely effective and robust in consistently defining dose delivery parameters across institutions using a variety of treatment planning and delivery systems for clinical trials,^40^ the same level of robustness has not been possible for tumor definitions. Algorithmic tools are an essential method for achieving this improved robustness.

Our tool illustrates a 26.9% improvement in segmentation agreement across physicians combined with a 20.3% improvement of agreement within the same physician contouring the same target. In addition, the tool reduced the time required for segmentation by 57.9% compared to the standard manual method per PET scan. In considering radiation therapy trials, this can impact both the cost by diminishing the numbers of patients needed and decreasing the time for defining the tumor target for treatment. Additional tools to apply these algorithmic benefits for response assessment will be both useful for outcome determination as well as potentially for therapy adaptation.

## 6. CONCLUSIONS

We have presented a novel approach for the highly computer-aided segmentation of lesions in H&N FDG PET volume data sets. By utilizing the JEI paradigm, a good trade-off between automation and input required from human experts was achieved, enabling segmentation of simple and complex cases with the same tool in an efficient and consistent manner. Due to the higher intra- and interoperator segmentation agreement compared to manual segmentation without a loss in segmentation accuracy, the proposed method is well suited for applications like image-guided radiation treatment planning as well as image based assessment of treatment response or treatment outcome prediction, which all benefit from these traits. The application of these tools in multi-institutional clinical trials can improve their efficiency and cost-effectiveness.

## Figures and Tables

**FIG. 15.**
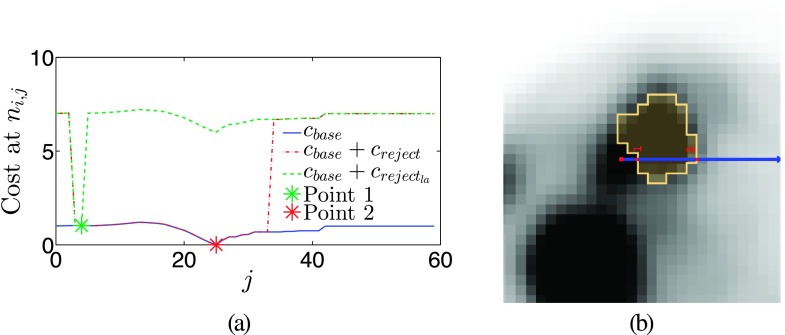
The effect of label avoidance on the cost function *c*. (a) Costs due to label avoidance along an axis, from *ce_k_*. Both *c*_reject_ and *c*_reject_*la*__ reject the first few nodes, but *c*_reject_*la*__ also begins to reject as soon as it encounters the other object label. The two points indicate the different low points in the cost function. (b) The axis on which the costs are changed with the same two points marked.

**FIG. 16.**
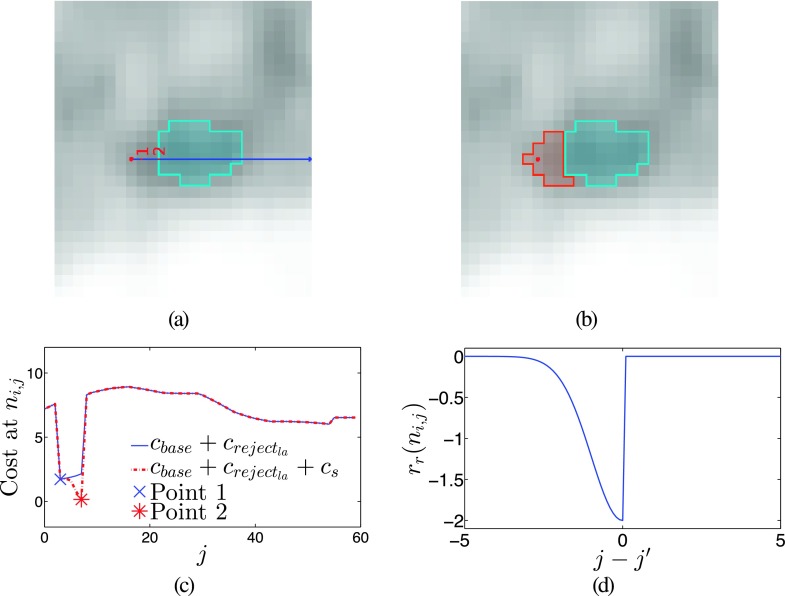
An example of a lesion with no real feature between its center and an adjacent lesion. (a) The graph center *ce_k_* and the other lesion in light blue. (b) The segmentation showing the sealing effect of label avoidance. (c) The cost change due to *c_s_* for the marked axis. (d) The general shape of the cost change. (See color online version.)

**FIG. 17.**
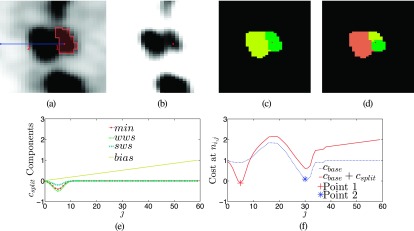
Segmenting a hot lymph node in close proximity to another hot node. (a) A segmentation produced in splitting mode with a single node column marked in blue. (b) Image as in (a), but with adjusted grey-value transfer function to better show the separation between the nodes. (c) Strong and (d) weak watersheds with *ce_k_* marked in red. Note that voxels with an uptake below *Th* are blacked out, since they do not affect the segmentation. (e) Additional cost components due to activated splitting option. (f) All components are added together with the base cost, resulting in the modified cost term; the corresponding segmentation is shown in (a). (See color online version.)

**FIG. 18.**
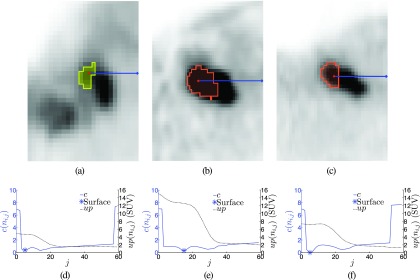
Examples of segmentations generated by using the splitting mode option. [(a)–(c)] Segmentations with one node column highlighted in blue. [(d)–(f)] Uptake and cost profiles corresponding to (a)–(c).

**TABLE I.** Overview of graph-based segmentation methods utilized for PET image segmentation.

Bagci *et al.* (Refs. 28 and 29)	Random-walk-based segmentation of lesions in PET images
Bagci *et al.* (Ref. 29)	Random-walk-based cosegmentation of PET and volumes produced with other imaging modalities
Ballangan *et al.* (Ref. 30)	Graph-cuts-based segmentation of lung cancer in PET images
Han *et al.* (Ref. 31) and Song *et al.* (Ref. 32)	Cosegmentation of single lesions in PET and CT images utilizing a Markov Random Field (MRF) approach; the optimization is solved using a graph-cuts-based method

**TABLE II.** Utilized PET-CT scanners and image reconstruction parameters.

PET-CT scanner	Number	Voxel size (mm)	Reconstruction algorithm
Siemens Biograph 40	13	3.394 × 3.394 × 2.025	2D OSEM, 4 iterations,
	2	3.394 × 3.394 × 5.000	8 subsets and a 7 mm Gaussian filter
Siemens Biograph Duo	1	3.432 × 3.432 × 3.375	2D OSEM, 2 iterations,
	43	3.538 × 3.538 × 3.375	8 subsets and a 5 mm Gaussian filter
GE Medical Systems Discovery LS	1	4.297 × 4.297 × 4.250	2D OSEM, 2 iterations, 28 subsets

**TABLE III.** Estimated mean Dice coefficient, intraoperator variability, and interoperator variability of segmentation accuracy.

	Manual		Semiautomated	
Measure	Value	95% CI	Value	95% CI
Dice coefficient mean	0.764	(0.741, 0.786)	0.766	(0.718, 0.815)
Intraoperator standard deviation	0.044	(0.0419, 0.0452)	0.037	(0.0359, 0.0387)
Interoperator standard deviation	0.049	(0.0462, 0.0513)	0.043	(0.0410, 0.0456)

**TABLE IV.** Estimated mean Dice coefficients for intra and interoperator segmentation agreement.

	Manual		Semiautomated	
Agreement	Mean	95% CI	Mean	95% CI
Intraoperator	0.770	(0.747, 0.794)	0.926	(0.902, 0.949)
Interoperator	0.713	(0.699, 0.727)	0.905	(0.891, 0.919)

**TABLE V.** Estimated mean times in minutes with standard deviation (SD) and 95% confidence intervals (CIs) for manual and semiautomated segmentations per PET data set.

Method	Mean	SD	95% CI
Manual	8.88	7.1	(6.47, 11.28)
Semiautomated	3.74	3.3	(2.40, 5.08)

**TABLE VI.** Statistics of actions required by expert 1 to expert 3 for the semiautomated method. All actions denotes the number of actions actually performed by the expert, and final actions denotes the number of actions that would have been required to generate the segmentations (i.e., undone actions are not counted).

		Number of actions		
Expert	Actions	Mean	Median	For 90%
1	All	1.57	1	3
2	All	2.43	1	6
3	All	1.80	1	3
1	Final	1.36	1	2
2	Final	1.37	1	2
3	Final	1.18	1	2

## APPENDIX A: LABEL AVOIDANCE

When generating a segmentation *S_k_*, it can be problematic if existing segmentations with other segmentation labels are overwritten. In such cases, the label avoidance option can be used to prevent this. This option affects the graph generation, cost calculation, and segmentation phase. First, if recentering is utilized (Sec. 2.A), label avoidance prevents *ce_k_* from being placed on or adjacent to (6-neighborhood) a lesion label in *L* different from that nearest to *ce*_*k*_user__.

Second, label avoidance modifies *c*_reject_ [Eq. (3)] by converting it into *c*_reject_*la*__. The modification requires knowledge of the closest label to a given node, which is known as *l*_*i*,*j*_ for node *j* on column *i*. Suppose that the object label currently being applied (to indicate an object in *L*) is *l*_new_ and that the label for no object in *L* is *l_bg_*. If the object on a node in a column is some other label *l*′ not equal to those, then that node and others beyond it are rejected with label avoidance to avoid including already segmented objects. For this purpose, a new condition *r*_3_(*n*_*i*,*j*_) = *j* > *j*_min_ and (∃*j*′ ∣ 0 ≤ *j*′ ≤ *j* and *l*_*i*,*j*′_ ≠ *l_bg_* and *l*_*i*,*j*′_ ≠ *l*_new_) is introduced in the if-clause of Eq. (3) with a logical OR operation. This prevents placing the boundary on an object in the existing labels image *L*. An example of label avoidance is shown in Fig. 15.

Third, if there is never a decrease in cost from the center point until the node that is part of another lesion label (Fig. 16), which will likely result in a gap between two objects that should be bordering to each other, then an additional cost term *c_s_*(*n*_*i*,*j*_) is added to *c*(*n*_*i*,*j*_). The cost term

$$c_{s}\left(n_{i,j}\right)=\{\begin{array}{ll} \mathrm{notch}\left(j,j^{\prime },2,1\right), & \text{if}\;\left(\exists j^{\prime }|csc\left(i,j^{\prime }\right)=\mathrm{True and}\;j\leq j^{\prime }\right)\\ 0, & \text{else} \end{array},$$ (A1)

with

$$\mathrm{notch}\left(j,j^{\prime },d,\sigma \right)=-d\;e^{\frac{-{\left(j-j^{\prime }\right)}^{2}}{2\sigma^{2}}}$$ (A2)

uses the cost seal condition *csc*(*n*_*i*,*j*_), which becomes *true* for a node *n*_*i*,*j*_ with *j* > *j*_min_ if it is adjacent to a different object label and the uptake is monotonically increasing between *j*_min_ − 1 and *j*. In addition, only the first occurrence of such a pattern is taken into account. For locations where *csc* becomes *true*, *c_s_* induces a cost minimum as shown in Fig. 16.

## APPENDIX B: SPLITTING

To facilitate the segmentation of an object with uptake in close proximity to an another object with similar uptake (e.g., hot lymph node that is part of an active lymph node chain), a splitting mode is implemented (Fig. 17). First, the soft smoothness constraint is increased from *sp* = 0.005 to *sp* = 0.05. Consequently, parts of a surface that are further away from the rest of the shape are more likely to be cut off. Furthermore, the surface becomes more responsive to the local refinement presented in Sec. 2.E.2. Second, the cost function *c* is expanded by adding *c*_split_, introducing additional feature terms that focus on local and potentially weak evidence of the presence of an uptake boundary. For this purpose, the following features are considered.

1. **Local minima:** Nodes representing local uptake minima along columns are detected by using *spc*_min_(*n*_*i*,*j*_) = (*up*(*n*_*i*,*j*−1_) > *up*(*n*_*i*,*j*_)) AND (*up*(*n*_*i*,*j*_) ≤ *up*(*n*_*i*,*j*+1_)).
2. **Watersheds:** For watershed analysis, an inverted version of image *I* is utilized.
  (i) Strong watersheds [Fig. 17(c)] are identified by a watershed segmentation with a fill level of 20% of the maximum difference of the processed volume, where the level is the threshold for watershed unions.^41^ The resulting watershed labels at node *n*_*i*,*j*_ will be denoted as *sws*(*n*_*i*,*j*_). Then, if two adjacent nodes on a column are in different watersheds and are in the above-threshold part of the column, the node is marked: *spc_sws_*(*n*_*i*,*j*_) = (*sws*(*n*_*i*,*j*−1_) ≠ *sws*(*n*_*i*,*j*_) and (∄*j*′ ∣ *up*(*n*_*i*,*j*′_) < *Th* and 0 ≤ *j*′ ≤ *j*)).
  (ii) Weak watersheds [Fig. 17(d)] are found by a watershed segmentation with a fill level of 0% of the maximum, which will likely result in oversegmentation. Nodes representing transitions between weak watersheds are marked by *spc_wws_*(*n*_*i*,*j*_) = (*wws*(*n*_*i*,*j*−1_) ≠ *wws*(*n*_*i*,*j*_) and (∄*j*′ ∣ *up*(*n*_*i*,*j*′_) < *Th* and 0 ≤ *j*′ ≤ *j*)). Note that weak watersheds will also respond to strong watersheds, but not vice versa.

Each node at which a splitting condition is met is a splitting node. Note that several markers described above can be set for a node on a given column. For each column, sets of nodes with the features *snl*_min_*i*__, *snl*_*sws_i_*_, and *snl*_*wws_i_*_ are combined into a superset *snl_i_*, containing all nodes that respond to corresponding features on column *i*.

At the location of a node with a condition met, a cost change is applied. For this purpose the notch function [Eq. (A2)] is utilized. This allows for the splitting feature to affect the surface solution, even if smoothness constraints prevent the exact splitting node from being part of the surface. The depth *d* is adjusted based on the type of splitting feature: $c_{{\mathrm{split}}_{\min}}\left(i,j\right)=\underset{\underset{i,\overset{\prime }{j}}{n}\in snl_{\min_{i}}}{\sum }\mathrm{notch}\left(j,j^{\prime },0.4,2\right)$, $c_{{\mathrm{split}}_{wws}}\left(i,j\right)=\underset{\underset{i,\overset{\prime }{j}}{n}\in snl_{wws_{i}}}{\sum }\mathrm{notch}\left(j,j^{\prime },0.5,2\right)$, and $c_{{\mathrm{split}}_{sws}}\left(i,j\right)=\underset{\underset{i,\overset{\prime }{j}}{n}\in snl_{sws_{i}}}{\sum }\mathrm{notch}\left(j,j^{\prime },0.2,2\right)$. Note that *d_sws_* < *d_wws_*, but weak watershed features usually also occur at nodes with strong watersheds, and thus their effects are additive, resulting in a cost reduction (i.e., notch depth) of 0.7 at such locations.

The additive cost term can now be defined as

$$c_{\mathrm{split}}\left(i,j\right)=\{\begin{array}{ll} c_{{\mathrm{split}}_{\min}}\left(i,j\right)+c_{{\mathrm{split}}_{wws}}\left(i,j\right)+c_{{\mathrm{split}}_{sws}}\left(i,j\right)+\frac{j+1}{n_{\mathrm{node}}} & \text{if}\;snl_{i}\neq 0̸,\\ 0, & \text{otherwise} \end{array}$$ (B1)

where the term (*j* + 1)/*n*_node_ represents a linear bias toward the center-most features of columns with splitting features. This equation is shown in its various parts in Figs. 17(e) and 17(f). The term *c*_split_ is added to *c*_base_ function, resulting in the final cost function *c*, which is used for calculating an optimal solution. The effect of this and the change to the soft smoothness constraint, when splitting is active, cause the method to be far more effective at segmenting lymph nodes in close proximity. Several examples of this mode’s effect are shown in Fig. 18.

## APPENDIX C: NECROTIC MODE

Necrotic lesions have a core with minimal or no uptake that is typically surrounded by active regions with uptake values above background. With the above presented framework, it will be difficult to segment such a necrotic lesion when the user places the center in the necrotic region. The main reason is that the approach presented above assumes that lesions have above background uptake values. This behavior can be changed by activating the necrotic mode option—instead of rejecting as soon as the uptake goes below the regional median, the uptake must first go above the threshold. Thus, the rejection for being too low will not happen immediately on necrotic regions, allowing the segmentation to succeed. For complex, very heterogeneous cases, a combination of normal and necrotic modes can be used to segment different dominant parts, which can be joined by assigning the same segmentation label (i.e., performing a logical OR operation).

## References

[1] J. W. Fletcher, B. Djulbegovic, H. P. Soares, B. A. Siegel, V. J. Lowe, G. H. Lyman, R. E. Coleman, R. Wahl, J. C. Paschold, N. Avril, L. H. Einhorn, W. W. Suh, D. Samson, D. Delbeke, M. Gorman, and A. F. Shields, “Recommendations on the use of 18F-FDG PET in oncology,” J. Nucl. Med. 49, 480–508 (2008). 10.2967/jnumed.107.047787 18287273

[2] C. R. Leemans, B. J. M. Braakhuis, and R. H. Brakenhoff, “The molecular biology of head and neck cancer,” Nat. Rev. Cancer 11, 9–22 (2011). 10.1038/nrc2982 21160525

[3] M. L. Gillison, “Human papillomavirus-associated head and neck cancer is a distinct epidemiologic, clinical, and molecular entity,” Semin. Oncol. 31, 744–754 (2004). 10.1053/j.seminoncol.2004.09.011 15599852

[4] S. B. Edge and A. J. C. on Cancer, AJCC Cancer Staging Manual, 7th ed. (Springer, New York, 2010), pp. xiv, 648 p.

[5] P. M. Som, H. D. Curtin, and A. A. Mancuso, “Imaging-based nodal classification for evaluation of neck metastatic adenopathy,” Am. J. Roentgenol. 174, 837–844 (2000). 10.2214/ajr.174.3.1740837 10701636

[6] M. Friedman, V. K. Shelton, M. Mafee, P. Bellity, V. Grybauskas, and E. Skolnik, “Metastatic neck disease: Evaluation by computed tomography,” Arch. Otolaryngol. 110, 443–447 (1984). 10.1001/archotol.1984.00800330025005 6732585

[7] A. A. Mancuso, H. R. Harnsberger, A. S. Muraki, and M. H. Stevens, “Computed tomography of cervical and retropharyngeal lymph nodes: Normal anatomy, variants of normal, and applications in staging head and neck cancer. Part II: Pathology,” Radiology 148, 715–723 (1983). 10.1148/radiology.148.3.6878692 6878692

[8] M. H. Stevens, H. Harnsberger, A. A. Mancuso, R. Davis, L. P. Johnson, and J. L. Parkin, “Computed tomography of cervical lymph nodes: Staging and management of head and neck cancer,” Arch. Otolaryngol. 111, 735–739 (1985). 10.1001/archotol.1985.00800130067007 4051864

[9] Y. Menda and M. M. Graham, “Update on 18F-fluorodeoxyglucose/positron emission tomography and positron emission tomography/computed tomography imaging of squamous head and neck cancers,” Semin. Nucl. Med. 35, 214–219 (2005). 10.1053/j.semnuclmed.2005.05.001 16150243

[10] Y. Menda and M. M. Graham, “FDG PET imaging of head and neck cancers,” in Positron Emission Tomography, edited byJuweid M. E. and Hoekstra O. S., Methods in Molecular Biology Vol. 727 (Humana, Totowa, NJ, 2011), pp. 21–31. 10.1007/978-1-61779-062-1_2 21331926

[11] The Eastern Cooperative Oncology Group (ECOG) and the American College of Radiology Imaging Network (ACRIN), “A multicenter trial of FDG-PET/CT staging of head and neck cancer and its impact on the N0 neck surgical treatment in head and neck cancer patients,” 2016, available at http://ecog-acrin.org/clinical-trials/acrin-6685-educational-materials.

[12] K. Pak, G. J. Cheon, H. Y. Nam, S. J. Kim, K. W. Kang, J. K. Chung, E. E. Kim, and D. S. Lee, “Prognostic value of metabolic tumor volume and total lesion glycolysis in head and neck cancer: A systematic review and meta-analysis,” J. Nucl. Med. 55, 884–890 (2014). 10.2967/jnumed.113.133801 24752671

[13] L. K. Shankar, J. M. Hoffman, S. Bacharach, M. M. Graham, J. Karp, A. A. Lammertsma, S. Larson, D. A. Mankoff, B. A. Siegel, A. Van den Abbeele, J. Yap, D. Sullivan, and I. National Cancer, “Consensus recommendations for the use of 18F-FDG PET as an indicator of therapeutic response in patients in national cancer institute trials,” J. Nucl. Med. 47(6), 1059–1066 (2006), available at see http://jnm.snmjournals.org/content/47/6/1059.long. 16741317

[14] R. L. Wahl, H. Jacene, Y. Kasamon, and M. A. Lodge, “From RECIST to PERCIST: Evolving considerations for PET response criteria in solid tumors,” J. Nucl. Med. 50, 122S–150S (2009). 10.2967/jnumed.108.057307 19403881 PMC2755245

[15] P. Tylski, S. Stute, N. Grotus, K. Doyeux, S. Hapdey, I. Gardin, B. Vanderlinden, and I. Buvat, “Comparative assessment of methods for estimating tumor volume and standardized uptake value in (18)F-FDG PET,” J. Nucl. Med. 51, 268–276 (2010). 10.2967/jnumed.109.066241 20080896

[16] E. Day, J. Betler, D. Parda, B. Reitz, A. Kirichenko, S. Mohammadi, and M. Miften, “A region growing method for tumor volume segmentation on PET images for rectal and anal cancer patients,” Med. Phys. 36, 4349–4358 (2009). 10.1118/1.3213099 19928065

[17] H. Li, W. L. Thorstad, K. J. Biehl, R. Laforest, Y. Su, K. I. Shoghi, E. D. Donnelly, D. A. Low, and W. Lu, “A novel PET tumor delineation method based on adaptive region-growing and dual-front active contours,” Med. Phys. 35, 3711–3721 (2008). 10.1118/1.2956713 18777930 PMC3304493

[18] R. J. McGurk, J. Bowsher, J. A. Lee, and S. K. Das, “Combining multiple FDG-PET radiotherapy target segmentation methods to reduce the effect of variable performance of individual segmentation methods,” Med. Phys. 40, 042501 (9pp.) (2013). 10.1118/1.4793721 23556917 PMC3612113

[19] X. Geets, J. A. Lee, A. Bol, M. Lonneux, and V. Gregoire, “A gradient-based method for segmenting FDG-PET images: Methodology and validation,” Eur. J. Nucl. Med. Mol. Imaging 34, 1427–1438 (2007). 10.1007/s00259-006-0363-4 17431616

[20] M. Hatt, C. C. le Rest, A. Turzo, C. Roux, and D. Visvikis, “A fuzzy locally adaptive Bayesian segmentation approach for volume determination in PET,” IEEE Trans. Med. Imaging 28, 881–893 (2009). 10.1109/TMI.2008.2012036 19150782 PMC2912931

[21] B. Foster, U. Bagci, A. Mansoor, Z. Xu, and D. J. Mollura, “A review on segmentation of positron emission tomography images,” Comput. Biol. Med. 50, 76–96 (2014). 10.1016/j.compbiomed.2014.04.014 24845019 PMC4060809

[22] S. Zhou, Medical Image Recognition, Segmentation and Parsing, Machine Learning and Multiple Object Approaches (Academic Press, London, 2015).

[23] R. Beichel, A. Bornik, C. Bauer, and E. Sorantin, “Liver segmentation in contrast enhanced CT data using graph cuts and interactive 3D segmentation refinement methods,” Med. Phys. 39, 1361–1373 (2012). 10.1118/1.3682171 22380370 PMC4109564

[24] R. R. Beichel and Y. Wang, “Computer-aided lymph node segmentation in volumetric CT data,” Med. Phys. 39, 5419–5428 (2012). 10.1118/1.4742845 22957609 PMC3432102

[25] S. Sun, M. Sonka, and R. R. Beichel, “Lung segmentation refinement based on optimal surface finding utilizing a hybrid desktop/virtual reality user interface,” Comput. Med. Imaging Graphics 37, 15–27 (2013). 10.1016/j.compmedimag.2013.01.003 PMC3852918 23415254

[26] S. Sun, M. Sonka, and R. Beichel, “Graph-based 4D lung segmentation in CT images with expert-guided computer-aided refinement,” in *2013 IEEE 10th International Symposium on Biomedical Imaging (ISBI)* (IEEE, 2013), pp. 1312–1315. 10.1109/ISBI.2013.6556773

[27] S. Sun, M. Sonka, and R. R. Beichel, “Graph-based IVUS segmentation with efficient computer-aided refinement,” IEEE Trans. Med. Imaging 32, 1536–1549 (2013). 10.1109/TMI.2013.2260763 23649180 PMC3883441

[28] U. Bagci, J. Yao, J. Caban, E. Turkbey, O. Aras, and D. Mollura, “A graph-theoretic approach for segmentation of PET images,” in *Annual International Conference of the IEEE Engineering in Medicine and Biology Society, EMBC* (IEEE, 2011), pp. 8479–8482. 10.1109/IEMBS.2011.6092092 PMC3476045 22256316

[29] U. Bagci, J. Udupa, N. Mendhiratta, B. Foster, Z. Xu, J. Yao, X. Chen, and D. Mollura, “Joint segmentation of functional and anatomical images: Applications in quantification of lesions from PET, PET-CT, MRI-PET, and MRI-PET-CT images,” Med. Image Anal. 17, 929–945 (2013). 10.1016/j.media.2013.05.004 23837967 PMC3795997

[30] C. Ballangan, X. Wang, M. Fulham, S. Eberl, and D. D. Feng, “Lung tumor segmentation in PET images using graph cuts,” Comput. Methods Prog. Biomed. 109, 260–268 (2013). 10.1016/j.cmpb.2012.10.009 23146420

[31] D. Han, J. Bayouth, Q. Song, A. Taurani, M. Sonka, J. Buatti, and X. Wu, “Globally optimal tumor segmentation in PET-CT images: A graph-based co-segmentation method,” in Information Processing in Medical Imaging (Springer-Verlag, Berlin Heidelberg, 2011), pp. 245–256. 10.1007/978-3-642-22092-0_21 PMC3158679 21761661

[32] Q. Song, J. Bai, D. Han, S. Bhatia, W. Sun, W. Rockey, J. Bayouth, J. Buatti, and X. Wu, “Optimal co-segmentation of tumor in PET-CT images with context information,” IEEE Trans. Med. Imaging 32, 1685–1697 (2013). 10.1109/TMI.2013.2263388 23693127 PMC3965345

[33] Y. Yin, X. Zhang, R. Williams, X. Wu, D. D. Anderson, and M. Sonka, “LOGISMOS–layered optimal graph image segmentation of multiple objects and surfaces: Cartilage segmentation in the knee joint,” IEEE Trans. Med. Imaging 29, 2023–2037 (2010). 10.1109/TMI.2010.2058861 20643602 PMC3131162

[34] K. Li, X. Wu, D. Z. Chen, and M. Sonka, “Optimal surface segmentation in volumetric images—A graph-theoretic approach,” IEEE Trans. Pattern Anal. Mach. Intell. 28, 119–134 (2006). 10.1109/TPAMI.2006.19 16402624 PMC2646122

[35] Q. Song, X. Wu, Y. Liu, M. Smith, J. Bautti, and M. Sonka, “Optimal graph search segmentation using arc-weighted graph for simultaneous surface detection of bladder and prostate,” in Proceedings of Medical Image Computing and Computer Assisted Intervention (MICCAI) (Springer-Verlag, Berlin Heidelberg, 2009), Vol. 5762, pp. 827–835. 10.1007/978-3-642-04271-3_100 20426188

[36] Y. Boykov and M.-P. Jolly, “Interactive graph cuts for optimal boundary and region segmentation of objects in N-D images,” in *Proceedings of International Conference on Computer Vision* (IEEE, 2001), Vol. 1, pp. 105–112.

[c37] 37.See www.slicer.org/slicerWiki/index.php/Documentation/4.5/Extensions/PETTumorSegmentation for more information about the current implementation of our software for 3D Slicer 4.5.

[38] S. K. Warfield, K. H. Zou, and W. M. Wells, “Simultaneous truth and performance level estimation (STAPLE): An algorithm for the validation of image segmentation,” IEEE Trans. Med. Imaging 23, 903–921 (2004). 10.1109/TMI.2004.828354 15250643 PMC1283110

[39] N. E. Makris, M. C. Huisman, P. E. Kinahan, A. A. Lammertsma, and R. Boellaard, “Evaluation of strategies towards harmonization of FDG PET/CT studies in multicentre trials: Comparison of scanner validation phantoms and data analysis procedures,” Eur. J. Nucl. Med. Mol. Imaging 40, 1507–1515 (2013). 10.1007/s00259-013-2465-0 23754762 PMC6704482

[40] G. S. Ibbott, A. Haworth, and D. S. Followill, “Quality assurance for clinical trials,” Front. Oncol. 3, 311 (11pp.) (2013). 10.3389/fonc.2013.00311 24392352 PMC3867736

[41] S. Lefèvre, “Knowledge from markers in watershed segmentation,” in *Proceedings of the 12th International Conference on Computer Analysis of Images and Patterns, CAIP’07* (Springer-Verlag, Berlin Heidelberg, 2007), pp. 579–586.

